# Supplementary material for: Analysis of PPARγ Signaling Activity in Psoriasis
Source: Int J Mol Sci. 2021 Aug 10;22(16):8603. doi: 10.3390/ijms22168603 (PMC8395241; doi:10.3390/ijms22168603)
Supplement: Supplementary file 1 [file ijms-22-08603-s001.zip › Supplemental materials_Analysis of PPARg signaling activity in psoriasis/Pathway models/Models images and html files/PPARG expressed in cells assotiated with psoriasis/225525.html]

regulatory T-cell


# CellType regulatory T-cell

|  |  |
| --- | --- |
| URN | urn:agi-ncimcelltype:C0039198 |
| Total Entities | 69 |
| Connectivity | 5502 |
| Name | regulatory T-cell |

---

|  |  |
| --- | --- |
| ChildConcepts | suppressor T-cell differentiation |
|  | CD8+ regulatory T-cell |
|  | effector/activated regulatory T-cell |
|  | extralymphoid regulatory T-cell |
|  | highly suppressive regulatory T-cell |
|  | Qa-1 restricted regulatory T-cell |
|  | CD4+ CD25- FOXP3+ T-cell |
|  | inducible CD4+CD25+ regulatory T-cell |
|  | memory regulatory T-cell |
|  | naive regulatory T-cell |
|  | CD4+ CD25+ regulatory T-cell |
|  | Tr1 cell |
|  | suppressor-inducer T-cell |
|  | regulatory T-cell homing |
|  | regulatory T-cell selection |
|  | regulatory T-cell homeostasis |
|  | regulatory T-cell development |
|  | regulatory T-cell adhesion |
|  | regulatory T-cell proliferation |
|  | regulatory T-cell count |
|  | regulatory T-cell survival |
|  | regulatory T-cell phenotype |
|  | regulatory T-cell function |
|  | regulatory T-cell migration |
|  | regulatory T-cell population |
|  | regulatory T-cell formation |
|  | regulatory T-cell size |
|  | regulatory T-cell polarity |
|  | regulatory T-cell death |
|  | regulatory T-cell repertoire |
|  | regulatory T-cell infiltration |
|  | regulatory T-cell apoptosis |
|  | regulatory T-cell priming |
|  | regulatory T-cell fate commitment |
|  | regulatory T-cell chemotaxis |
|  | regulatory T-cell clonal anergy |
|  | regulatory T-cell fate |
|  | regulatory T-cell localization |
|  | regulatory T-cell regeneration |
|  | regulatory T-cell interaction |
|  | regulatory T-cell proliferative response |
|  | regulatory T-cell ratio |
|  | regulatory T-cell sequestration |
|  | regulatory T-cell transendothelial migration |
|  | regulatory T-cell viability |
|  | regulatory T-cell motility |
|  | regulatory T-cell division |
|  | regulatory T-cell growth |
|  | regulatory T-cell compartmentalization |
|  | regulatory T-cell extravasation |
|  | regulatory T-cell spreading |
|  | regulatory T-cell distribution |
|  | regulatory T-cell fate determination |
|  | regulatory T-cell contact |
|  | regulatory T-cell disruption |
|  | regulatory T-cell rolling |
|  | regulatory T-cell autophagy |
|  | regulatory T-cell renewal |
|  | regulatory T-cell damage |
|  | regulatory T-cell cytotoxicity |
|  | regulatory T-cell destruction |
|  | regulatory T-cell aggregation |
|  | regulatory T-cell volume |
|  | regulatory T-cell motion |
|  | regulatory T-cell invasion |
|  | regulatory T-cell transdifferentiation |
|  | regulatory T-cell phagocytosis |
|  | regulatory T-cell mediated cytotoxicity |
|  | regulatory T-cell communication |

---

|  |  |
| --- | --- |
| Pathway | Tumor Infiltrating Macrophages in Cancer Progression and Immune Escape |
|  | Myeloid Derived Suppressor Cells in Cancer Immune Escape |
|  | IDO1 in Cancer Immune Escape |
|  | CD Markers -> Expression Targets in Lymphoid System and Blood |
|  | PPARG expressed in cells assotiated with psoriasis |
|  | Overview. Diseases of the skin (chapter 11) |
|  | 2\_Defective tolerance of autoreactive T-cell in T1D |

---

|  |  |
| --- | --- |
| MedScan ID | 10004137 |

---

|  |  |
| --- | --- |
| Alias | suppressor T lymphocyte |
|  | T suppressor cells |
|  | regulatory T-lymphocyte |
|  | suppressor T-cells |
|  | Treg cells |
|  | T suppressor lymphocytes |
|  | Tregs |
|  | Treg |
|  | regulatory T-cell |
|  | T cell regulatory |
|  | T suppressor cell |
|  | Treg cell |
|  | T regulatory cell |
|  | regulatory T-lymphocytes |
|  | suppressor T-cell |
|  | immunoregulatory T-lymphocyte |
|  | T regulatory cells |
|  | T suppressor lymphocyte |
|  | immunoregulatory T-lymphocytes |
|  | T-lymphocytes, regulatory |
|  | T regulatory lymphocytes |
|  | suppressor T lymphocytes |
|  | T cells regulatory |
|  | T lymphocyte, regulatory |
|  | immunoregulatory T-cell |
|  | regulatory T-cells |
|  | immunoregulatory T-cells |
|  | T regulatory lymphocyte |

---

|  |  |
| --- | --- |
| NCIm ID | C0039198 |

---
